# A signal amplification assay for HSV type 1 viral DNA detection using nanoparticles and direct acoustic profiling

**DOI:** 10.1186/1477-3155-8-3

**Published:** 2010-02-14

**Authors:** Yildiz Uludağ, Richard Hammond, Matthew A Cooper

**Affiliations:** 1 Cranfield Health, Cranfield University, Cranfield, Bedfordshire, MK43 0AL, UK; 2 Cambridge Medical Innovations, 181 Cambridge Science Park, Cambridge, CB4 0GJ, UK; 3 Institute for Molecular Bioscience, University of Queensland, 306 Carmody Rd., St Lucia, Qld 4072, Australia

## Abstract

**Background:**

Nucleic acid based recognition of viral sequences can be used together with label-free biosensors to provide rapid, accurate confirmation of viral infection. To enhance detection sensitivity, gold nanoparticles can be employed with mass-sensitive acoustic biosensors (such as a quartz crystal microbalance) by either hybridising nanoparticle-oligonucleotide conjugates to complimentary surface-immobilised ssDNA probes on the sensor, or by using biotin-tagged target oligonucleotides bound to avidin-modified nanoparticles on the sensor. We have evaluated and refined these signal amplification assays for the detection from specific DNA sequences of Herpes Simplex Virus (HSV) type 1 and defined detection limits with a 16.5 MHz fundamental frequency thickness shear mode acoustic biosensor.

**Results:**

In the study the performance of semi-homogeneous and homogeneous assay formats (suited to rapid, single step tests) were evaluated utilising different diameter gold nanoparticles at varying DNA concentrations. Mathematical models were built to understand the effects of mass transport in the flow cell, the binding kinetics of targets to nanoparticles in solution, the packing geometries of targets on the nanoparticle, the packing of nanoparticles on the sensor surface and the effect of surface shear stiffness on the response of the acoustic sensor. This lead to the selection of optimised 15 nm nanoparticles that could be used with a 6 minute total assay time to achieve a limit of detection sensitivity of 5.2 × 10^-12 ^M. Larger diameter nanoparticles gave poorer limits of detection than smaller particles. The limit of detection was three orders of magnitude lower than that observed using a hybridisation assay without nanoparticle signal amplification.

**Conclusions:**

An analytical model was developed to determine optimal nanoparticle diameter, concentration and probe density, which allowed efficient and rapid optimisation of assay parameters. Numerical analysis and subsequent associated experimental data suggests that the response of the mass sensitive biosensor system used in conjunction with captured particles was affected by i) the coupled mass of the particle, ii) the proximal contact area between the particle and the sensor surface and iii) the available capture area on the particle and binding dynamics to this capture area. The latter two effects had more impact on the detection limit of the system than any potential enhancement due to added mass from a larger nanoparticle.

## Background

The detection of pathogen-specific nucleic acid sequences provides a precise and accurate method for clinical and environmental screening. Real-time, label-free biosensors have the potential to provide rapid and precise detection of nucleic acids, provided that sample preparation (including nucleic acid extraction) is accomplished without user intervention, and the requisite sensitivity and specificity for detection is achieved. As a label-free method, quartz crystal microbalance (QCM) technology provides a rapid and effective method for the detection of both protein analytes (antigen immunoassays) and nucleic acid testing (NAT). The frequency change of a QCM biosensor can be described in terms of the total mass of the bound molecules, associated shear modulus imparted by the bound analyte layer and the non-binding bulk viscosity and density changes of the liquid adjacent to the sensor surface [1]. Inclusion of additional mass in the form of nanoparticles conjugated to a specific sequence recognition element enables the detection of significantly lower concentrations of DNA or RNA fragments.

There are two principle ways in which nanoparticles are used for NAT enhancement. In the first method, nanoparticles are conjugated to target oligonucleotides that hybridise to the probe on the sensor surface [2-4]. In the second method, biotin tagged target oligonucleotides bind to avidin-modified nanoparticles [4-7]. The latter scheme is relatively simple to implement since avidin modified nanoparticles can be used for different DNA sequence detection assays, whereas the former method requires specific oligonucleotide modified nanoparticles for individual assays. Additionally the assay can be performed either as homogeneous or heterogeneous assay formats [4,8-12]. For example Mao *et al*. used streptavidin modified ferric oxide nanoparticles (ca. 145 nm diameter) for the detection of *Escherichia coli *O157:H7 [5]. By employing a heterogeneous assay format with a 10 minute hybridisation period followed by a 10 minute signal enhancement with nanoparticles under flow, they achieved a detection limit of 10^-12 ^M for synthetic DNA sequences. Pang *et al*. employed DNA probe-modified 13 nm gold nanoparticles to detect specific sequences from the β-thalassemia gene [13]. By means of a heterogeneous assay and one hour hybridisation at 55°C in a static cell followed by a further hour incubation with nanoparticles, they achieved a detection limit of 2.6 × 10^-9 ^M. Liu *et al*. modified a QCM sensor surface with gold nanoparticles to increase the available surface capture area, then enhanced the hybridisation signal with gold nanoparticles derivatised with thiolated complimentary DNA [14]. In this case, the hybridisation assay was performed for two hours at 40°C in a static cell with a resultant a detection limit of 10^-16 ^M. Whilst these assay formats can deliver impressive limits of detection, they suffer from long incubation times and/or complex amplification procedures requiring multiple steps that are not suited to a rapid, point of care test format.

In the current study, we describe the detection of specific, conserved DNA sequences of herpes simplex virus (HSV) type 1. HSV causes recurrent mucosal infections of the eye, mouth and genital tract. HSV type 1 establishes a lifelong latent infection within the host which can subsequently reactivate to cause recurrent infections and occasionally life threatening HSV encephalitis. The probe and complementary target sequence used for the HSV recognition assays was from VP16 gene region of HSV viral sequence, which encodes for an essential structural protein and also functions as a major virion trans-activator of virus gene expression [15]. HSV regulatory protein VP16 plays key roles to stimulate viral gene expression during the earliest stages of infection, thus it is relevant to diagnose clinical HSV infection by detecting the genes encoding VP16 as this is an important replication and virulence determinant.

The objective of this study was to investigate the optimal methodology for signal enhancement with gold nanoparticles to enable both sensitive *and *rapid HSV viral sequence detection. In our previous study [16] we observed that a semi-homogeneous assay format (in which probe and complimentary target are pre-mixed in solution) led to a lower assay detection limit than a heterogeneous, two-step flow-based assay. Completely homogeneous assays are advantageous in that they allow single step, rapid tests that require minimal amounts of sample and are easier to embody in a device suitable for point-of-care diagnostic testing. In this study the results of semi-homogeneous and completely homogeneous assays were compared for both NeutrAvidin (NA) and NA-modified gold nanoparticle signal enhancement methods. An analytical model for the optimal nanoparticle diameter, concentration and probe density was developed to allow selection of a sub-set of subsequent experimental conditions for evaluation.

## Materials and methods

Resonant acoustic profiling (RAP) experiments were conducted using an automated four-channel RAP ◆ id 4 instrument (RAP ◆ id 4; TTP Labtech, Royston, UK). The instrument applies the principles of QCM sensing, in that a high frequency (16.5 MHz) oscillating voltage is applied to a piezoelectric quartz crystal to induce the crystal to resonate, and its resonance frequency is then monitored in real time. RAP ◆ id 4 integrates acoustic detection with a continuous flow micro fluidic delivery system, a thermal control unit, and automated sample handling. Four individual flow cells enable up to four measurements to be performed simultaneously. The volume of each flow cell used in this study was 900 nl. The time required to exchange the complete volume of the flow cell could be set as low as 2.2 seconds at a flow rate of 25 μl/min and as high as 14 milliseconds at a flow rate of 4000 μl/min. In order to minimise sample consumption, 25 μl/min was employed for the pathfinder assay development. Baseline drift observed during the study was 0.25 ± 0.15 Hz (n = 12) after docking and priming the sensor chips. The operating temperature was 25 ± 0.5°C throughout the assays.

### Preparation of NeutrAvidin modified gold nanoparticles

NeutrAvidin modified gold nanoparticles were synthesized by derivatizing 1 ml of aqueous gold nanoparticles (BBInternational, Cardiff, UK) with 6 μl of a 1 mg/ml solution of NA. The mixture was incubated for an hour on a shaker at room temperature. Then 100 μl of 10 mg/ml BSA was added and allowed to stand on a shaker for further 20 minutes, followed by centrifugation to remove excess reagents. The supernatant was removed; then 33 μl 10 mg/ml BSA, 100 μl Tris buffer (20 mM Tris-HCl, 150 mM NaCl, 1 mM EDTA, pH 7) and 1 μl of 5% sodium azide were added. The modified gold nanoparticles were stored at 4°C and warmed to room temperature before use.

### Sensor Surface Preparation

AKT ◆ *iv *Covalent sensor chips (TTP Labtech, Royston, UK) were employed for the assays. Sensor surfaces were prepared by immobilising NeutrAvidin (NA; Perbio Science UK Ltd, Cramlington, UK) on sensors using conventional amine coupling chemistry. The running buffer used for immobilisation was degassed Dulbecco's modified phosphate buffered saline (PBS, pH 7.4; Sigma-Aldrich, Poole, UK). The flow rate of the buffer for the assay was 25 μl/min. Sensor surfaces were first activated with a 1:1 mixture of 400 mM EDC and 100 mM NHS (LINK ◆ *it *Coupling Solution kit; TTP Labtech, Royston, UK), prepared in 0.22 μm-filtered deionised water, and mixed immediately prior to use (final concentrations; 200 mM EDC and 50 mM NHS). EDC-NHS was injected simultaneously across all four sensor surfaces for 3 minutes. NA (50 μg/mL in PBS buffer) was then injected simultaneously across sensor surfaces for 3 minutes. Non-reacted NHS esters were capped with 1 M ethanolamine, pH 8.5 (LINK ◆ *it *Coupling Solution kit; TTP Labtech, Royston, UK). Frequency changes relating to protein coupling were recorded 2 minutes after the protein injection was completed. After NA immobilisation, the running buffer was changed to Tris buffer comprising 20 mM Tris-HCl, 150 mM NaCl, 1 mM EDTA, pH 7. Biotinylated complementary surface probe and scrambled surface probe (biotinylated probes; TIB Molbiol, Berlin, Germany; Table 1) were diluted in Tris buffer to 10 μg/ml and injected separately over different flow cells for 3 minutes to create active and control surfaces. The frequency changes of the biotinylated probes captured were recorded 4 minutes after the end of the injection.

**Table 1:** Nomenclatures and sequences of HSV type 1 and control oligonucleotides.

Name	DNA Sequence
VP16 Surface probe	5'-Biotin- CTC GTT GGC GCG CTG AAG CAG GTT TTT G-3' -3'
VP16 Scrambled surface probe	5'-Biotin-ACC TGG GCA TGT ATG GTG TCG TCG CGT T-3' -3'
VP16 Target sequence	5'-AAA ACT TCC GTA CCC CT CA A AA A CC T GC T TC A-3'
VP16 Detection probe	5'-GGG TAC GGA AGT TTT TCA CTC GAC - Biotin-3'

### Hybridisation Signal Enhancement Assay

Running buffer used for the assay was Tris buffer comprising 20 mM Tris-HCl, 150 mM NaCl, 1 mM EDTA, 0.05% Tween 20, pH 7. Initially 10 mM Biotin (Sigma-Aldrich, Poole, UK) in Tris buffer was injected for 1 minute to block the remaining active sites of the NA layer then semi-homogeneous and homogeneous assays were performed for VP16 target detection.

#### Semi-homogeneous assay

**Figure 1:**
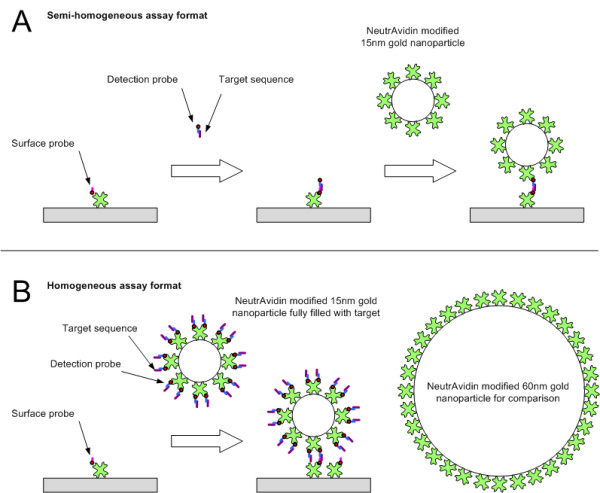
**Schematic of the assay formats for semi- homogeneous (A) and homogeneous (B) hybridisation signal enhancement assay**.

The VP16 target sequence and VP16 detection probe were hybridised in a tube at 55°C for 3 minutes at required concentrations; VP16 detection probe concentration being at least twofold higher concentration than the VP16 target sequence concentration. The resultant hybridised material was then injected over the sensor surface to be captured by VP16 surface probe. Subsequently, to increase the signal NA or NA modified gold nanoparticle solutions were injected for 3 minutes (Figure 1 - A). The frequency change due to the binding was recorded 180 seconds after the injection started.

#### Homogeneous assay

The VP16 target sequence and VP16 detection probe were hybridised at 55°C for 3 minutes at required concentrations; VP16 detection probe concentration being at least twofold higher than the VP16 target sequence concentration. Depending on the assay evaluated either NA or NA modified gold nanoparticles was then added to the hybridised VP16 target and detection probe solution. Subsequently this mixture was injected across the sensor surface coated with surface probe as described above (Figure 1 - B). The frequency change was recorded 180 seconds after the beginning of the injection.

## Results and Discussion

### HSV-VP16 hybridisation and signal enhancement assay

50 μg/ml NA was immobilised to AKT ◆ *iv *Covalent sensors, then 10 μg/ml biotinylated probe was captured on all sensor surfaces. Biotinylated DNA capture on the NA layer resulted in 235 ± 10 Hz response (n = 8, data not shown), followed by capping of the remaining NA biotin binding sites with 10 mM biotin. As a control surface VP16 scrambled sequences were captured on the NA layer and this was followed by the injection of NA modified gold nanoparticle solution. The level of non-specific binding observed following exposure to of 7 × 10^11 ^particles/ml 15 nm NA modified gold nanoparticles, 3 × 10^10 ^particles/ml 40 nm or 60 nm NA modified gold nanoparticles was 1 ± 1 Hz (n = 7, data not shown).

### Homogeneous and semi-homogeneous assay with NA

In our previous work [16] we have found that DNA hybridisation efficiency could be higher when hybridisation was performed at annealing temperature in free solution rather than via *in situ *hybridization to a probe on the biosensor surface. The conditions of hybridisation are a key assay component that defines the stringency of hybridisation [17]. Two of the most important components of hybridisation conditions are salt concentration and temperature; high stringency is favoured by low salt concentrations and high temperatures, which together promote the hybridisation of perfectly matched single stranded nucleotides to form double stranded sequences. It is more practical and appropriate to vary the annealing temperature of a homogenous solution before injection than vary the temperature of the flowing solution and biosensor; in addition the hybridisation process in the bulk, 3D, solution will be more rapid than that which would occur at the planar, 2D sensor surface. Before embarking on a comparative study of reagent ratios, assay formats and particle properties, calculations were performed to assess the expected effect of various assay components on signal evolution.

Firstly the maximum possible number of NA molecules on the flow cell surface was estimated by modelling the NA molecules as spheres packed onto the flat flow cell surface area with an assumed packing density of 0.907 (the circle packing density limit) [18]. The number of NA molecules can be described by:(1)

Where a_F _is the flow cell area (12.5 mm^2^) and r_NA _is the radius of the NA molecule (3 nm). The immobilisation of the surface NA was carried out at 3.3 × 10^-7 ^M concentration using 75 μl of solution (1.49 × 10^13 ^molecules); to achieve the saturation of the surface calculated in equation (1) above 2.7% of the material in solution has to reach the flow cell surface. This value is the required mass transport efficiency of the flow cell (that is the ratio of the mass of material reaching the surface to the mass of material entering the flow cell) to achieve sensor surface saturation.

To estimate the actual mass transport efficiency of the flow cell a time-stepping mathematical model of the flow cell was built. The model is a two-dimensional representation of a cross-section through the flow cell above the sensor surface; the inputs include the flow velocity of the liquid matrix, the binding kinetics of the NA molecules to the surface (using a Langmuir adsorption model) and the diffusion properties of particles through the flow cell to the surface driven by the concentration gradient created by particles binding at the surface. This simulation suggests the flow cell mass transport efficiency is approximately 1% for the conditions used for NA immobilisation in this work. Assuming an efficiency of 1% for the flow cell, the actual number of NA attached to the flow cell surface during immobilisation is approximately 1.5 × 10^11 ^molecules. The system is mass transport limited and the sensor surface does not reach saturation of NA molecules. Finally the active part of the sensor has a surface area of 3.14 mm^2^, one-quarter of the flow cell surface; hence the number of NA molecules on the active sensor can be estimated as 3.7 × 10^10^.

This calculated 1% mass transport efficiency noted above is used for all subsequent analysis. The mass transport efficiency is affected by four key parameters: the surface availability (number of binding sites on the surface), diffusion characteristics of the transported species within the liquid, the binding kinetics of the species to the surface and the initial concentration of species in the liquid passed through the flow cell. Measurements of transport efficiency of the flow cell geometry used in these experiments have been made [unpublished data] using antigen binding to antibodies on the sensor surface (i.e. a similar size species to the NA used in these experiments but a lower affinity binding mechanism at the surface). This data indicates the flow cell efficiency to be between 0.1% and 1% with the higher efficiencies observed at lower analyte input concentrations. Given the well-known difficulties of measuring the transport efficiency of a flow cell accurately and the number of variables that affect the efficiency a nominal 1% mass transport efficiency has been used throughout to simplify the analysis.

The number of probes binding to the surface NA can be estimated assuming an average of two probes bind per NA (out of the four available sites only two, on average, are accessible [19,20]. Assuming this 2:1 ratio the number of probes required to saturate the flow cell NA surface is 3 × 10^11^. When 75 μl of probe is injected at a concentration of 1.1 × 10^-6 ^M this implies a total of 5 × 10^13 ^potential hybridizations. To achieve surface saturation, only 0.6% of these probes need to reach the flow cell surface, a figure within the efficiency estimate for the flow cell geometry used. Hence the number of probes on the sensor surface can be estimated to be 7.4 × 10^10^, which is twice the number of NA molecules.

In the semi-homogenous assay, 75 μl of VP16 target was injected at 5.2 × 10^-10 ^M. Assuming 1% mass transport efficiency this suggests that 5.8 × 10^7 ^targets will reach the sensor surface to bind. This is over a thousand-fold less than the number of probes present on the surface; thus the target is expected to be relatively sparsely distributed across the sensor surface with an average predicted spacing of approximately 260 nm. When 75 μl of NA at 8.3 × 10^-8 ^M (5 μg/ml) is injected, this reagent is in excess again. Given the targets are, on average, spatially very distant compared to the size of the NA molecules we would expect only one NA to bind per target. Thus the number of NA on the surface at the completion of the semi-homogeneous assay can be estimated to be 5.8 × 10^7^. In contrast, for the homogeneous assay the target and NA are pre-mixed before injection into the flow cell. At the same final molar concentrations as the semi-homogenous assay (5.2 × 10^-10 ^M and 8.3 × 10^-8 ^M respectively) the NA is in excess. Assuming immediate, homogeneity between the two volumes, the number of targets binding per NA molecule will follow the Poisson probability distribution (equation 2):(2)

where p(x) is the probability of x targets binding per NA molecule and μ is the mean number of targets per molecule, i.e. the ratio of molarities. In this case μ is very low (0.006) so most NA have no targets, a small proportion have one target and almost none have two or more targets. Hence with this assumption we would expect evolved signals on the sensor to be the result of single target-NA interactions, the same as the semi-homogenous format.

We recall that using the previously reported [16] semi-homogeneous assay with NA enhancement of signal, the detection limit obtained for the VP16 probe was 5.2 × 10^-11 ^M. When the semi-homogeneous and homogeneous assay formats were compared experimentally for detection of 5.2 × 10^-10 ^M VP16 target (10 times the detection limit), the homogeneous assay resulted in a signal of 10 ± 4 Hz (n = 2) and the semi-homogeneous assay resulted in a signal of 25 ± 3 Hz (n = 4). The measured homogeneous assay response was half the response for the semi-homogeneous format suggesting only half the quantity of NA binds to the surface in the homogenous format - that is, for the same concentration of target two targets are binding per NA molecule and thus 2.9 × 10^7 ^NA molecules are bound to the surface. This is not as predicted using the Poisson distribution model assuming complete homogeneity in the first mixing of target and NA for the homogeneous assay format.

**Figure 2:**
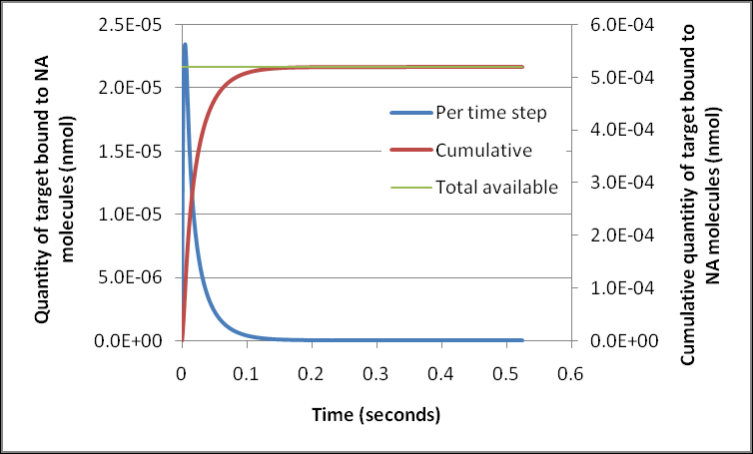
**Estimated binding kinetics of target (5.2 × 10^-10 ^M concentration) to NA molecule using a Langmuir binding model K_a _= 10^15 ^M^-1^**. Curves show instantaneous (blue) and cumulative (red) quantities of target bound.

Looking again at the mixing of the homogeneous solution, by implementing a simple Langmuir adsorption model of target to the NA molecule the rate of complex creation can be estimated. For the high k_a _value (on rate) for the biotin-neutravidin system (7.06 × 10^7 ^M^-1^.s^-1^) [21] the model suggests the NA molecules introduced into the target solution become bound with all the available target in approximately 0.2 of a second (Figure 2). This speed of binding is much faster than the NA injection time into the target solution suggesting the homogeneous format allows more targets to bind per NA molecule than the semi-homogenous format because of the favourable binding kinetics in the three-dimensional space of the bulk solution, leading to a lower signal from the sensor.

In conclusion, once packing density, stoichiometry *and *varying reaction kinetics imparted by the dimensionality of hybridisation are taken into account, we would not expect to improve the sensitivity of the DNA hybridisation assay using NA amplification alone in the absence of nanoparticles. The key issues are the low mass of the NA molecules and their small radius; when multiple targets bind to the NA only one of them can be brought into proximity with the surface to make a bond. This suggests the assay performance may be increased by using more massive, larger diameter amplification particles such as gold nanoparticles.

### Homogeneous and semi-homogeneous assay with nanoparticle enhancement

Again, before embarking on a comparative study of reagent ratios, assay formats and particle properties, calculations were performed to assess the expected effect of nanoparticle size on signal evolution. Three diameters of particles were chosen for analysis: 60, 40 and 15 nm with a respective mass ratio of 64: 19: 1.

For the semi-homogeneous assay, assuming the same performance of the surface NA binding and probe binding as for the previous calculation, 75 μl of target at 1.4 × 10^-9 ^M with a 1% mass transport efficiency gives an estimated 1.6 × 10^8 ^targets on the sensor surface, on average 160 nm apart. The number of NA molecules on the surface of the gold nanoparticle can be estimated in the same way as the sensor surface by modelling them as packed spheres. For a 60 nm particle, ~360 NA molecules are required to pack the surface completely; the conjugation conditions with excess NA ensure the particles are fully packed (Table 2). These fully-packed particles are then injected into the flow cell, 75 μl at 3.0 × 10^10 ^particles per ml. At 1% mass transport efficiency this indicates 5.6 × 10^6 ^gold particles reach the sensor surface. These approximate calculations of average target and particle surface densities indicate the surface is very sparsely populated with material. There is, on average, one target every 160 nm along the surface and one gold particle for every 28 targets. Under these sparse conditions it is reasonable to assume only one target will bind to each gold particle; it is geometrically difficult for multiple bonds to form between the surface and the nanoparticles.

**Table 2:** Number of NA molecules available for an individual gold nanoparticle.

Number of NA molecules used to modify gold nanoparticles	Gold nanoparticle diameter (nm)	Number of gold nanoparticles in 1 ml solution used for modification*	NA capacity of each nanoparticle when excess NA molecules used
6.02 × 10^13^	15	1.4 × 10^12^	23
6.02 × 10^13^	40	8.9 × 10^10^	161
6.02 × 10^13^	60	2.6 × 10^10^	363

*Determined from the suppliers BBInternational (Cardiff, UK).

Finally, we consider the signal evolution of the bound nanoparticles through the piezoelectric QCM biosensor. The most widely used formula for predicting frequency shift in an acoustic sensor under load is the Sauerbrey equation (equation 3) [22](3)

where ρ_s _is the surface mass density (mass per unit surface area). Applying this formula simplistically to this semi-homogenous assay format with a 16.5 MHz nominal fundamental frequency f_0 _indicates a frequency shift of -233 Hz from 5.6 × 10^6 ^60 nm gold particles. However this formula assumes a mechanically rigid, homogenous layer on the sensor surface; the reality of a sparse distribution of large particles attached to the surface by single NA chains does not approximate well to this model. In particular the surface is not rigid, hence we would not expect the response to match this prediction.

**Figure 3:**
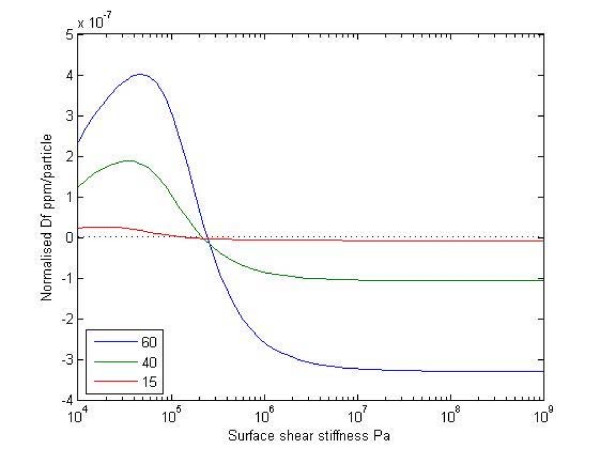
**Predicted change in sensor frequency (expressed as parts per million of the fundamental sensor frequency per particle bound) as a function of capture layer shear stiffness for 60, 40 and 15 nm gold particles for the 16.5 MHz sensor used in the experimental work**. Note the rapid loss of sensitivity as the stiffness drops below 1 × 10^6 ^Pa.

As the Sauerbrey model is not a good approximation to the actual surface, it is instructive to look more closely at the effect of surface stiffness on the sensor response. To do this a multi-layer acoustic wave mathematical model of the sensors was built [23]. Figure 3 shows the predicted sensor response as a function of shear stiffness of the bound layer and the gold particle size. At high stiffness (1 × 10^7 ^Pa and greater) the sensor shows a consistent negative frequency response - the Sauerbrey limit. As the surface becomes less stiff the response reduces significantly, actually passing through zero to become positive. This result indicates that the surface stiffness is a key characteristic of the sensor response, not just the mass attached to the surface. Increasing the number of bonds between the gold particles and the sensor surface will increase the stiffness and give a greater response per particle attached. A non-rigid layer as described above for the semi-homogenous assay format is expected to have significantly reduced response from the Sauerbrey limit.

In the case of the homogeneous assay we assume that immediate injection of the particles gives rise to a completely homogeneous solution the distribution of target per particle; this should follow the Poisson distribution with a mean of 32 (where the mean is the ratio of molarities at the same target concentration as before, 1.4 × 10^-9 ^M) and a standard deviation of 17. For a 60 nm particle there are ~730 target bind sites per particle assuming 2 targets can bind per NA as before. Hence the gold particles would, on average, be 4% full; the targets are far apart on the particles. However, from the previous results with NA alone, we know the assumption of a Poisson distribution is not a good one: in reality the relatively slow injection rate of particles into the target solution gives an inhomogeneous solution. Some particles are completely filled and others have no target at all. Using the same Langmuir binding kinetic model as for the NA enhanced assay estimates 10% of the 60 nm gold particles will be full of target and the other 90% have no target. When these filled particles pass across the probe-covered sensor surface multiple target and probe pairs are made proximal due to the relatively large radius of curvature of the particle: multiple bonds are made between each particle and the surface as the probes hybridise with the target. We know that surface stiffness is important for obtaining sensor sensitivity. These multiple bonds increase the stiffness significantly and thus are expected to give a greater negative frequency change signal. As the concentration of target reduces, the number of targets bound per particle reduces. At a target concentration of 5.2 × 10^-11^M the Langmuir binding kinetic model predicts only 8 targets per gold particle. The system is now not capable of making multiple particle-sensor surface bonds as the targets are widely spaced on the particles again.

In summary, the sensor response is dependent both on the mass of the particle and the stiffness of the connection between the particle and the sensor. Due the binding kinetics in 3D space a homogenous assay format creates particles with multiple targets allowing high avidity bonding between the particle and the surface. A semi-homogenous format only allows single bonds to take place between particle and surface. Thus for given concentrations of particles and analyte a homogenous format is expected to give significantly better response than a semi-homogenous format.

**Figure 4:**
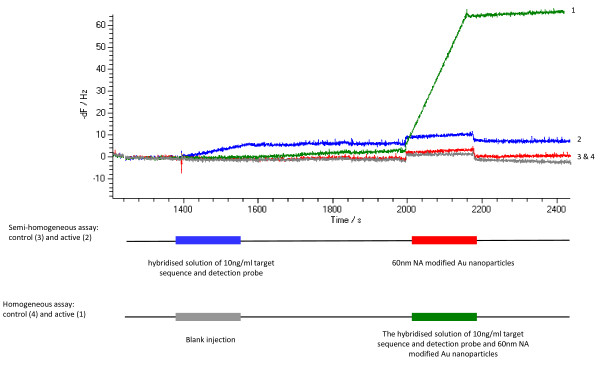
**Semi-homogeneous and homogeneous assays using 60 nm NA modified gold nanoparticles**.

Using the 60 nm particles, semi-homogeneous and homogeneous formats were assay experimentally using 1.4 × 10^-9 ^M (6.32 × 10^10 ^molecules in 75 μl) VP16 target and 3.0 × 10^10 ^particles/ml (2.3 × 10^9 ^particles in 75 μl) 60 nm NA modified gold nanoparticles. While 61 Hz of response was obtained with the homogeneous assay, no response was observed with the semi-homogeneous assay (Figure 4). This result confirmed the homogenous format was preferred with 60 nm gold particle enhancement as it allowed high avidity bonding to the surface giving strong acoustic coupling (bond stiffness). Lower concentrations of target were tested to probe the lower limit of detection. VP16 target at 5.2 × 10^-10 ^M led to a 35 Hz response, but no response was observed at 5.2 × 10^-11 ^M. This was consistent with the expected response based on volume binding kinetics analysis of the nanoparticles described above.

This analysis and experimental data suggests the response of a mass sensitive biosensor system used in conjunction with captured particles is affected by i) the coupled mass of the particle, ii) the proximal contact area between the particle and the sensor surface and iii) the available capture area on the particle and binding dynamics to this capture area. These latter two effects appear to have more impact on the detection limit of the system than any potential enhancement due to added mass from the larger particle. Experimentally, reducing the diameter of the nanoparticle from 60 nm to 40 nm did not result in any significant change in assay detection limit (data not shown), so the study was extended to use 15 nm diameter NA modified gold nanoparticles.

In theory, smaller particles should have two advantages: (i) for a given number of targets more particles are able to be bound during the homogeneous binding step so more mass can reach the sensor and (ii) for a given density of targets on the particle the targets are closer together and promote high-avidity coupling to the sensor surface though this is tempered by the smaller radius removing the targets from proximity to the surface. In the case of the 15 nm particle there are expected to be approximately 23 NA molecules per particle, equating to 46 biotin binding sites (Table 2). If 2.4 × 10^10 ^molecules of target are injected (5.2 × 10^-10^M) the Langmuir binding kinetic model suggests approximately 8% of the particles will be bound with 10 targets per particle. When this mixture is injected at a concentration of 3 × 10^10 ^particles/ml to the flow cell, assuming 1% mass transport efficiency as before, approximately 1.3 × 10^7 ^gold particles are able to bind tightly to the sensor surface with multiple bonds through the 10 targets. At the lower concentration of target (5.2 × 10^-11^M) the binding kinetics model suggests one target per particle on 8% of the particles. However, the same number of particles bind to the sensor but are less well coupled acoustically through a single bond - the reduced stiffness is expected to give a lower response.

**Figure 5:**
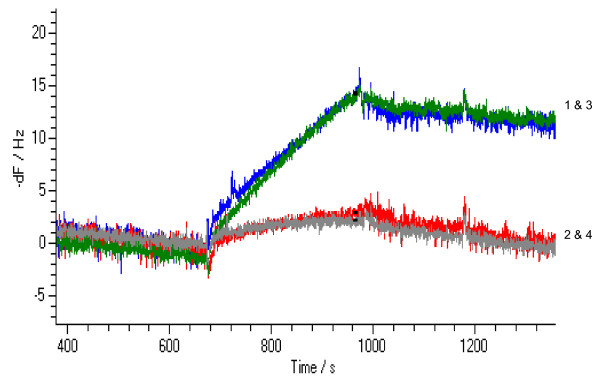
**Homogeneous assay with NA modified 15 nm gold nanoparticles for the detection of 5 × 10^-11 ^M target**. Traces 1 & 3 are responses on active channels; traces 2 & 4 are responses on control channels.

**Figure 6:**
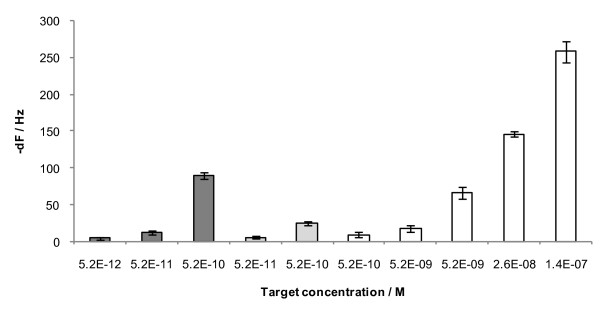
**Concentration vs frequency change plot for VP16 target hybridization to VP16 surface probe**. White: Heterogeneous assay with signal enhancement using NA. Light grey: Semi-homogeneous assay with signal enhancement with NA. Dark grey: Homogeneous assay and with 15 nm NA modified Au nanoparticles. Error bars represent standard deviations for n = 4.

When assayed experimentally, the 15 nm nanoparticle assay have a 89 ± 3 Hz signal at 5.2 × 10^-10 ^M target concentration and 12 ± 1 Hz signal at 5.2 × 10^-11 ^M target concentration using 5.25 × 10^10 ^gold nanoparticles (Figure 5). When the experiment was performed using lower concentrations of the target, it was possible to detect down to 5.2 × 10^-12 ^M of VP16 target, i.e. at a concentration 10 times lower than the signal enhancement assay with NA and with a signal response 1000 times higher than the direct assay without any signal enhancement (Figure 6).

To assess the quality of the homogeneous assay with NA modified 15 nm gold nanoparticles, Z-factor analysis was employed. The Z-factor provides an easy and useful measure for assay quality and has been a widely accepted standard. Z-factor reflects both the assay signal dynamic range and the data variation associated with the signal measurements; where a Z-factor between 0.5 and 1.0 is an excellent assay; between 0 and 0.5 is marginal, and less than 0 means that the signal from the positive and negative controls overlap, indicating the invalidity of the assay results (equation 3; average (μ) and standard deviation (σ) of both active and control DNA hybridisation results).(4)

Z-factor values were calculated for the hybridisation of 5.2 × 10^-11 ^M and 5.2 × 10^-10 ^M target sequences as 0.62 and 0.84, respectively, indicating good to excellent assay performance.

### Summary

In this study homogeneous and semi-homogenous assays were compared using both NA and NA modified gold nanoparticles, and the effect of particle size on amplification efficiency was investigated by use of 15 nm, 40 nm and 60 nm gold nanoparticles. The highest sensitivity was achieved with the homogeneous assay using 15 nm gold nanoparticles. To obtain good response from an acoustic sensor the target particles need to be strongly acoustically coupled. This is achieved by creating multiple bonds between particles arriving at the surface and the surface itself. For binding reactions with a high association constant making a homogenous solution of target and particle allows the assembly of a small number of densely-packed particles as the targets bind to the particles faster than the particles are added to the solution. For large particles this assembly process places much of the target on the 'wrong side' of the particle; the target cannot interact with a two-dimensional surface. With smaller particles the target is more advantageously distributed between particles allowing more material to bind strongly to the sensor surface. The interaction between target-particle binding kinetics and binding avidity to the sensor surface becomes increasingly critical as the quantity of target is reduced.

As can be seen from the examples given in the introduction section, the conditions of the hybridisation assay show great variation between applications. The length of the target DNA sequence (hence molecular weight of the ligand), hybridisation temperature, hybridisation time, assay format (static or flow, homogeneous or heterogeneous), the frequency of the quartz crystal and size of nanoparticles used are some of these variations and all of these contribute to the sensitivity of the QCM assays. Addition of mass in the form of nanoparticles improves the detection limit of the DNA hybridisation assays to the region of 10^-12 ^M. To lower detection limit, a further amplification can be applied by means of catalytic deposition of gold on to gold nanoparticles or catalytic precipitation by means of alkaline phosphatase [24,25]. Although it is possible to reach detection limit up to 10^-16 ^M [25] using catalytic precipitation methods, these reactions increase the assay time, inherently cause higher variability of results and would be more difficult to apply in a miniaturised, point-of-care device. Other methods applied include use of gold nanoparticles to enhance the immobilisation of the probe and then amplification with a second set of gold nanoparticles [10,26]. Studies are ongoing to lower the detection limit of the DNA hybridisation assay using such simplified procedures.

## Conclusion

In this paper we demonstrated a sensitive and rapid assay for the detection of HSV 1 viral sequences. With use of 15 nm gold nanoparticles and a 6 minutes assay time, three orders of magnitude lower sensitivity was obtained than the assay without nanoparticles amplification. There are several reports cited herein that describe empirical results using different sized nanoparticles for near identical or similar assays, and for which detection sensitivities are observed to vary by several orders of magnitude. However, to the best of our knowledge, this work is the first example of a detailed theoretical analysis towards a better understanding of the mechanisms that lead to such differences and the somewhat counterintuitive observation that a small diameter particle leads to greater sensitivity in a mass-based biosensor assay.

## Competing interests

The authors declare that they have no competing interests.

## Authors' contributions

YU carried out the experimental study, contributed to the analysis of the results and drafted the manuscript. RH participated in the analysis of the results and drafting of the manuscript. MAC conceived of the study, and participated in its design and coordination and helped to draft the manuscript. All authors contributed in the preparation of the manuscript.
